# Continuous Relationship of Operative Duration with Risk of Adverse Perioperative Outcomes and Early Discharge Undergoing Thoracoscopic Lung Cancer Surgery

**DOI:** 10.3390/cancers15020371

**Published:** 2023-01-06

**Authors:** Chaoyang Tong, Yaofeng Shen, Hongwei Zhu, Jijian Zheng, Yuanyuan Xu, Jingxiang Wu

**Affiliations:** 1 Department of Anesthesiology, Shanghai Chest Hospital, Shanghai Jiao Tong University, Shanghai 200052, China; 2 Department of Anesthesiology, Shanghai Children’s Medical Center, School of Medicine, Shanghai Jiao Tong University, Shanghai 200127, China; 3 Department of Lung Cancer Center, Shanghai Chest Hospital, Shanghai Jiao Tong University, Shanghai 200030, China

**Keywords:** thoracoscopic surgery, operative duration, lung cancer, outcomes

## Abstract

**Simple Summary:**

Operative duration is considered an important predictor of poor prognosis in several surgical specialties; however, for patients receiving thoracoscopic lung cancer surgery, whether this association remains, and what the trigger point of operative duration contributing to a dramatic increase in adverse perioperative outcomes (APOs) and a significant decrease in early discharge is, is unknown. This retrospective cohort study included 12,392 patients who underwent this surgical treatment and explored the continuous relationship of operative duration with risk of the six most common APOs and early discharge using restricted cubic spline (RCS). The results showed that the risk of APOs exponentially increases as the operative duration exceeds 90 min, accompanied by a significant decrease in the likelihood of early discharge, indicating that shortening the operative duration to less than 90 min may be an important adjustable factor to greatly reduce APOs and accelerate early discharge.

**Abstract:**

Background: For thoracoscopic lung cancer surgery, the continuous relationship and the trigger point of operative duration with a risk of adverse perioperative outcomes (APOs) and early discharge remain unknown. Methods: This study enrolled 12,392 patients who underwent this surgical treatment. Five groups were stratified by operative duration: <60 min, 60–120 min, 120–180 min, 180–240 min, and ≥240 min. APOs included intraoperative hypoxemia, delayed extubation, postoperative pulmonary complications (PPCs), prolonged air leakage (PAL), postoperative atrial fibrillation (POAF), and transfusion. A restricted cubic spline (RCS) plot was used to characterize the continuous relationship of operative duration with the risk of APOs and early discharge. Results: The risks of the aforementioned APOs increased with each additional hour after the first hour. A J-shaped association with APOs was observed, with a higher risk in those with prolonged operative duration compared with those with shorter values. However, the probability of early discharge decreased from 0.465 to 0.350, 0.217, and 0.227 for each additional hour of operative duration compared with counterparts (<60 min), showing an inverse J-shaped association. The 90 min procedure appears to be a tipping point for a sharp increase in APOs and a significant reduction in early discharge. Conclusions: Our findings have important and meaningful implications for risk predictions and clinical interventions, and early rehabilitation, for APOs.

## 1. Introduction

As part of a trend of minimally invasive surgery (MIS), thoracoscopic surgery has been increasingly used for pulmonary resection, which significantly reduces surgical stress, systemic inflammation, and postoperative complications [1,2,3]. It has also become the optimal treatment for primary lung cancer when combined with systemic lymph node dissection [4]. Despite the clear benefits of MIS, perioperative complications still remain high, such as hypoxemia [5], new-onset arrhythmia [6], delayed extubation [7], postoperative pulmonary complications (PPCs) [8,9], and prolonged air leakage (PAL) [10,11]. The reasons for this event are multifactorial, including specific comorbidities such as age-related impairment of lung function and tissue fragility [12], the unique physiology of one-lung ventilation (OLV) [13] (including hypoperfusion or reperfusion, and atelectasis or reinflation), and increased inflammatory mediators and postoperative incisional chest wall pain [14,15].

Many studies have identified preoperative comorbidities for surgical risk stratification [16,17,18], but none have assessed the impact of surgical factors, particularly operative time, on prognosis. Recently, a growing body of evidence has shown that operative duration was an independent and potentially modifiable risk factor for poor outcomes in multiple surgical specialties: bariatric, neurosurgical, esophagectomy, spine, urologic, and colectomy [19,20,21,22,23,24]. However, for patients undergoing thoracoscopic lung cancer surgery, the continuous effects of operative duration on adverse perioperative outcomes (APOs) and early discharge were not well focused on and understood [25,26]. Furthermore, the trigger point of operative duration contributing to a dramatic increase in APOs and a significant decrease in early discharge is unknown. Thus, this study aims to use the restricted cubic spline (RCS) functions for the first time to investigate the continuous relationship of operative duration with a risk of APOs and early discharge in this type of surgical treatment by reviewing a large number of prospectively collected data and to identify the optimal turning point.

## 2. Materials and Methods

### 2.1. Study Design and Patients

This cohort study was performed following the approval of the Institutional Review Board (IRB) of Shanghai Chest Hospital (chair: Dr. Zheng Ning, permission NO.IS22040) on 7 June 2022, with informed consent waived. Between March 2016 and December 2018, we reviewed the medical records of 14,149 consecutive patients who underwent thoracoscopic lung cancer surgery. The excluded patients were described in the flow diagram (Supplementary Figure S1). In total, 12,392 patients were enrolled in the final analysis. This article adheres to Strengthening the Reporting of Observational Studies in Epidemiology (STROBE) guidelines.

### 2.2. Anesthesia Protocol

All patients were routinely monitored by electrocardiogram, pulse oximetry, noninvasive blood pressure (NIBP), and capnography. Radial artery intubation and right internal jugular central venous catheterization (CVP) were used to monitor invasive blood pressure (IBP). After the anesthesia and muscle relaxation phase was completed, all patients received endotracheal intubation with a visual laryngoscope and a fiberoptic bronchoscope to ensure the correct placement of the tube. Thoracic paravertebral blockade (TPVB) was completed by giving 15 mL 0.5% ropivacaine through T4-T5 under the guidance of the GE ultrasound system using a high-frequency linear array probe by a designated anesthesiologist after induction. The intraoperative lung protective ventilation strategies consisted of low-tide ventilation based on ideal body weight (≤8 mL/kg), PEEP = 5 cm H_2_O, lung recruitment, and maintenance of airway pressure at <30 cm H_2_O. All patients received a patient-controlled analgesia (PCA) pump, including sufentanil 1.0 μg/kg + desoxocin 0.4 mg/kg.

### 2.3. Technique of Operation

Since 2016, the high-volume center of Shanghai Chest Hospital has completed nearly 10,000 lung operations each year, of which thoracoscopic surgery accounts for more than 80%. A total of 13 physicians served as chief surgeons, in charge of 13 surgical groups, and each group also had 1 to 2 surgical assistants. All thoracoscopic procedures were determined by the participating surgeons on the basis of the patient’s preoperative evaluation, operative planning, and surgical experience. Our published study has described the annual volume of surgeries performed by 13 chief surgeons [6,27], well beyond the learning curve [28], so we assumed that these chief surgeons have comparable technical abilities. Detailed approaches related to such surgical treatment could also be found in [27]. For all patients, anatomic lung resection plus systematic lymph node dissection (which included at least three mediastinal and hilar lymph nodes) was regarded as the optimal treatment for primary lung cancer.

### 2.4. Data Collection and APOs

Perioperative clinical data were prospectively extracted from our institution’s electronic medical records, including patient’s baseline characteristics, intraoperative variables (such as tumor size), advanced tumor stage, pleural adhesions, anesthesia type, type of resection, surgical procedure, location of resection, clinical nodal involvement, lymph nodes calcification, and APOs. APOs included intraoperative hypoxemia, delayed extubation, PPCs, PAL, postoperative atrial fibrillation (POAF), transfusion, and the length of hospital stay (LOS).

### 2.5. Definition

Hypoxemia was defined as SPO_2_ ≤ 90%, lasting for 5 min [5]. Extubation performed outside the operating room (OR) was defined as delayed extubation [29]. PPCs refer to the European Perioperative Clinical Outcome (EPCO) [30]. PAL was defined as gas leakage (air bubbles in chest drainage system after coughing or deep breathing) and failure to remove the chest drainage tube 5 days after surgery [10,11]. In accordance with 2014 American Association for Thoracic Surgery guidelines, POAF was defined as when the ECG recordings with features of AF lasting at least 30 s or requiring drug treatment or anticoagulation [31]. According to the LOS, early and routine discharge were defined as “discharge on postoperative day 2” and “discharge after postoperative day 2”, respectively [32]. Typically, increased operative duration was categorically defined relative to a cut point (e.g., <1 h or >1 h) or per minute(s) of surgery [26]. Additionally, in this cohort study, 5 groups were stratified by operative duration: <60 min, 60–120 min, 120–180 min, 180–240 min, and ≥240 min.

### 2.6. Statistical Analysis

Statistical power calculations were not performed prior to this study, because the sample size was based on available data in our data set. Statistics and data analysis plans were defined before accessing the data and were completed after the data had been accessed. Continuous variables were compared using one-way ANOVA or Kruskal–Wallis tests. Categorical variables were compared with the Chi-squares test or the Fisher exact test, depending on the sample size. A univariate analysis showed that all factors that were significantly correlated with APOs and early discharge (*p* < 0.2) were inserted into the multivariate logistic regression model using the forward selection strategy.

RCS functions that permitted nonlinear associations can be used to assess the inflection point at which the risk of a certain outcome changes; put simply, these functions allow researchers to circumvent the trap of arbitrary dichotomous continuous variables [33,34]. In this study, the continuous relationship of operative duration with a risk of APOs and early discharge were examined using RCS (OR and Log OR curves, with 3 knots), treating 90 min of operative duration as the reference with adjustments of the aforementioned covariates. The 95% CI was computed along a continuous spectrum of operative duration. We also performed sensitivity analyses to determine whether the association between operative duration (>90 min, vs. ≤90 min) and a risk of APOs differed by sex (male/female), age (≤65 years or >65 years), comorbidities (with/without), or anesthesia type (with/without TPVB), adjusting for the aforementioned values. A statistical analysis was performed with the SPSS 26.0 software (IBM Corp., Armonk, NY, USA). The R version 4.1.2 was used with the packages of tidyr, dplyr, forestplot, rms, and pROC. Lastly, *p*-value < 0.05 was considered statistically significant.

## 3. Results

### 3.1. Study Cohort

Baseline and intraoperative characteristics among five cohorts were presented in Table 1. In total, 12,392 patients receiving thoracoscopic lung cancer surgery were eventually included, of which 3.8% (470 out of 12,392) developed intraoperative hypoxemia, 1.5% (189 out of 12,392) developed delayed extubation, 33.2% (4115 out of 12,392) developed PPCs, 7.9% (984 out of 12,392) developed PAL, 2.7% (340 out of 12,392) developed POAF, 0.8% (98 out of 12,392) developed transfusion, and 16.7% (2072 out of 12,392) developed early discharge.

### 3.2. Incidence among Five Groups of APOs and Early Discharge

For intraoperative complications, the incidence of hypoxemia ranged from 26 out of 1336 (1.9%) to 284 out of 8365 (3.4%), to 134 out of 2322 (5.8%), to 22 out of 307 (7.2%), and to 4 out of 62 (6.5%) (*p* < 0.001) with the increase in operative duration. In terms of postoperative complications, the occurrence of delayed extubation increased from 12 out of 1336 (0.9%) to 101 out of 8365 (1.2%), to 57 out of 2322 (2.5%), to 12 out of 307 (3.9%), and to 7 out of 62 (11.3%) (*p* < 0.001) with the augment of operative duration. The occurrence of PPCs increased from 269 out of 1336 (20.1%) to 2727 out of 8365 (32.6%), to 948 out of 2322 (40.8%), to 138 out of 307 (45.0%), and to 33 out of 62 (53.2%) (*p* < 0.001) with the prolongation of operative duration.

In addition, the occurrence of PAL increased from 60 out of 1336 (4.5%) to 578 out of 8365 (6.9%), to 269 out of 2322 (11.6%), to 60 out of 307 (19.5%), and to 17 out of 62 (27.4%) (*p* < 0.001) with longer operative duration. Moreover, the occurrence of POAF increased from 12 out of 1336 (0.9%) to 207 out of 8365 (2.5%), to 87 out of 2322 (3.7%), to 24 out of 307 (7.8%), and to 10 out of 62 (16.1%) (*p* < 0.001) with prolonged operative duration. Additionally, the occurrence of transfusion increased from 5 out of 1336 (0.4%) to 43 out of 8365 (0.5%), to 47 out of 2322 (2.0%), to 20 out of 307 (6.5%), and to 11 out of 62 (17.7%) (*p* < 0.001) with an increase in operative duration. Finally, the occurrence of early discharge decreased from 435 out of 1336 (32.6%) to 1346 out of 8365 (16.1%), to 266 out of 2322 (11.5%), to 21 out of 307 (6.8%), to 4 out of 62 (6.5%) (*p* < 0.001) with an increase in surgical duration (Table 1).

### 3.3. Adjusted Odds Radio (aOR) among Groups (vs. <60 min)

Risks of the aforementioned APOs increased with each additional hour after the first hour. Further, aORs for intraoperative hypoxemia increased from 1.628 to 2.574, 3.111, and 2.618; delayed extubation, from 1.209 to 2.182, 2.932, and 8.727; PPCs, from 1.822 to 2.391, 2.666, and 3.631; PAL, from 1.366 to 1.987, 2.925, and 4.082; POAF, from 2.566 to 3.272, 5.647, and 12.839; and transfusion, from 1.350 to 4.838, 13.844, and 41.063 in groups with 60–120 min, 120–180 min, 180–240 min, and ≥240 min of operative duration, respectively. However, the likelihood of early discharge decreased from 0.465 to 0.350, 0.217, and 0.227 for each additional hour of operative duration when compared with their counterparts (<60 min) (Table 2).

### 3.4. OR and Log OR Plot in APOs and Early Discharge

**Figure 1:**
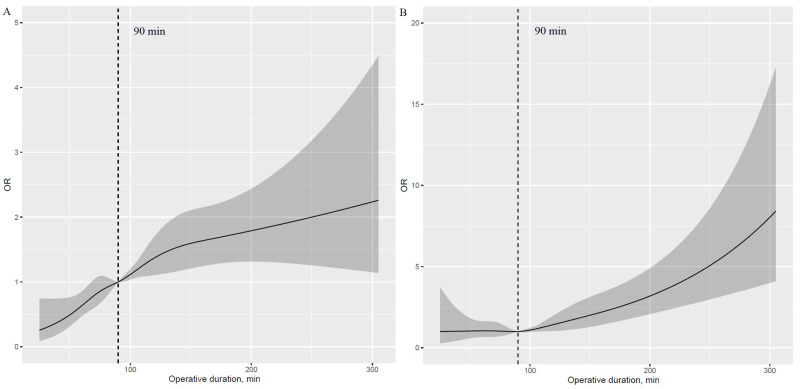

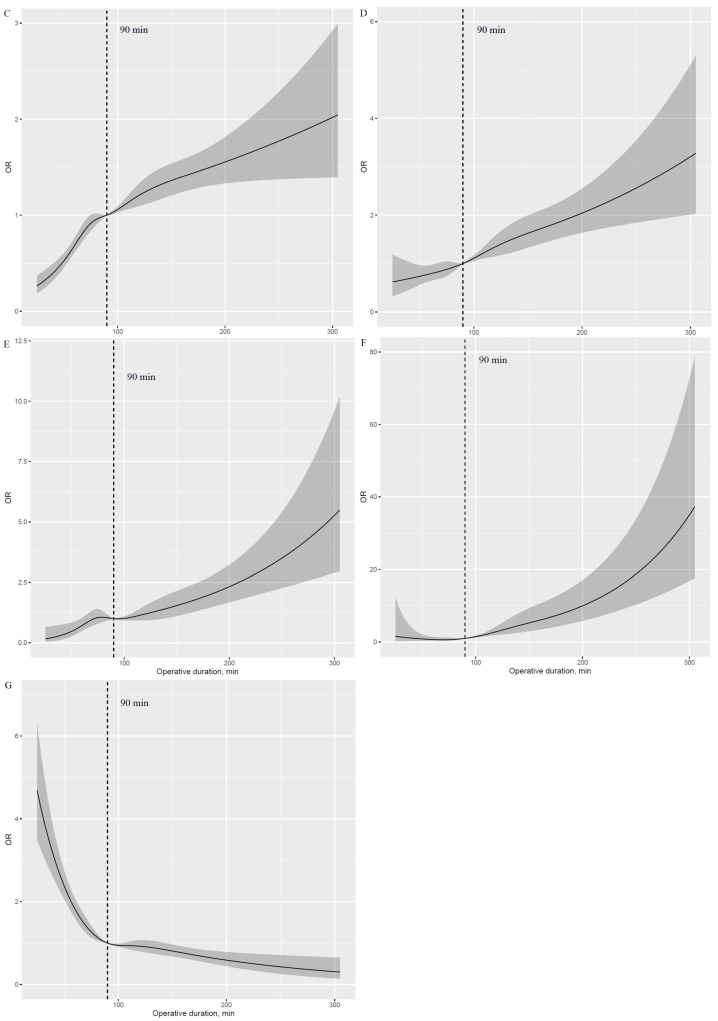
Nonlinear association of operative duration with risk of APOs and early discharge (HR plot) adjusted for covariates. (**A**): Nonlinear association between operative duration and risk of hypoxemia. (**B**): Nonlinear association between operative duration and risk of delayed extubation. (**C**): Nonlinear association between operative duration and risk of PPCs. (**D**): Nonlinear association between operative duration and risk of PAL. (**E**): Nonlinear association between operative duration and risk of POAF. (**F**): Nonlinear association between operative duration and risk of transfusion. (**G**): Nonlinear association between operative duration and risk of early discharge. APOs: adverse perioperative complications; PPCs: postoperative pulmonary complications; PAL: prolonged air leakage; POAF: postoperative atrial fibrillation.

**Figure 2:**
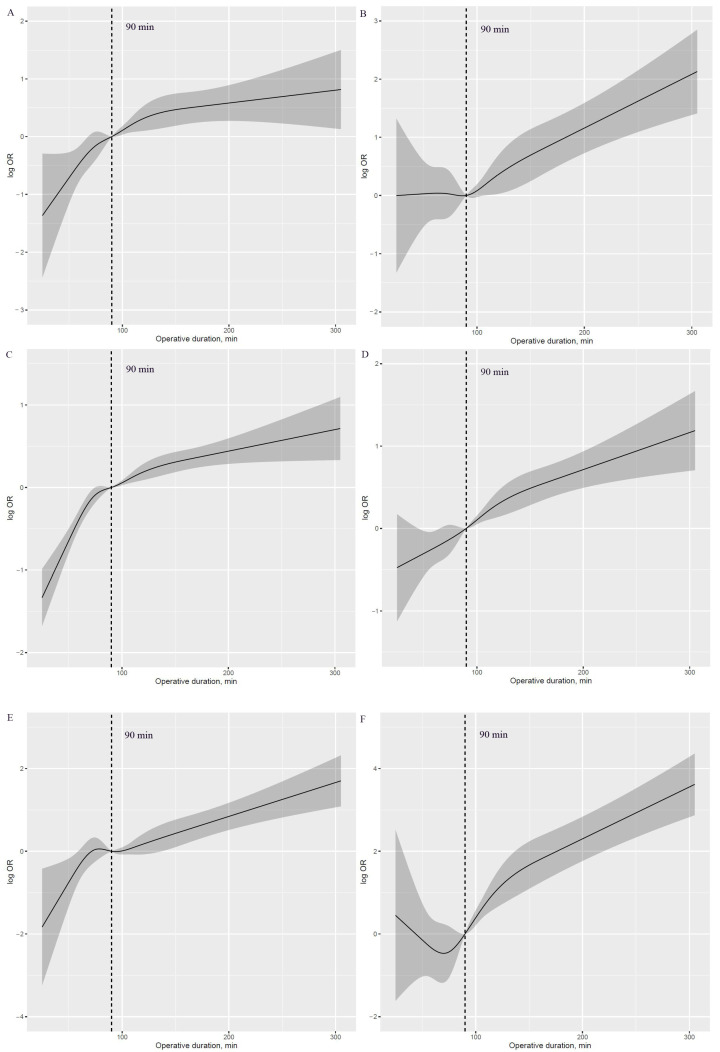

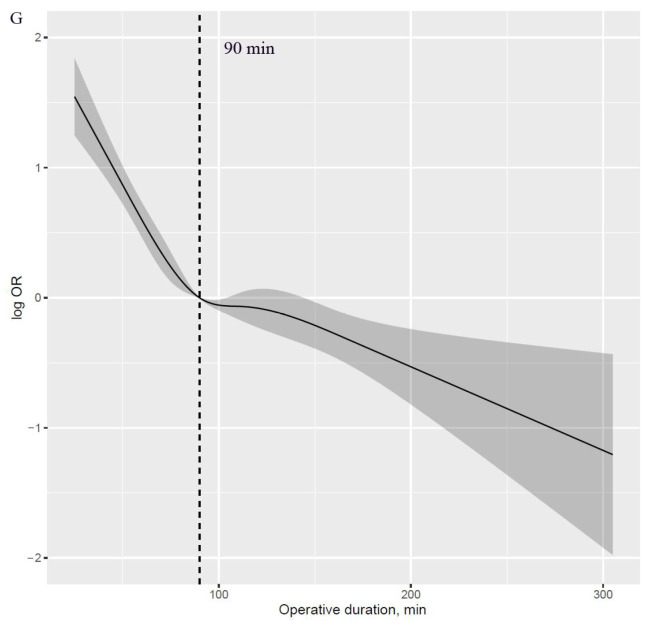
Log HR plot of operative duration with risk of APOs and early discharge, adjusted for covariates. (**A**): Log HR plot between operative duration and risk of hypoxemia. (**B**): Log HR plot between operative duration and risk of delayed extubation. (**C**): Log HR plot between operative duration and risk of PPCs. (**D**): Log HR plot between operative duration and risk of PAL. (**E**): Log HR plot between operative duration and risk of POAF. (**F**): Log HR plot between operative duration and risk of transfusion. (**G**): Log HR plot between operative duration and risk of early discharge. APOs: adverse perioperative complications; PPCs: postoperative pulmonary complications; PAL: prolonged air leakage; POAF: postoperative atrial fibrillation.

A J-shaped association with APOs was observed with higher risk in those with prolonged operative duration compared with those with shorter values (Figure 1A–F). Additionally, an inverse J-shaped association with early discharge was observed, indicating that patients with shorter operative duration were more likely to be discharged early than those with longer values (Figure 1G). The 90 min procedure appears to be a tipping point for a dramatic increase in APOs and a significant reduction in the likelihood of early discharge (Figure 2A–G).

### 3.5. Forest Plot for APOs and Early Discharge in Sensitivity Analyses

**Figure 3:**
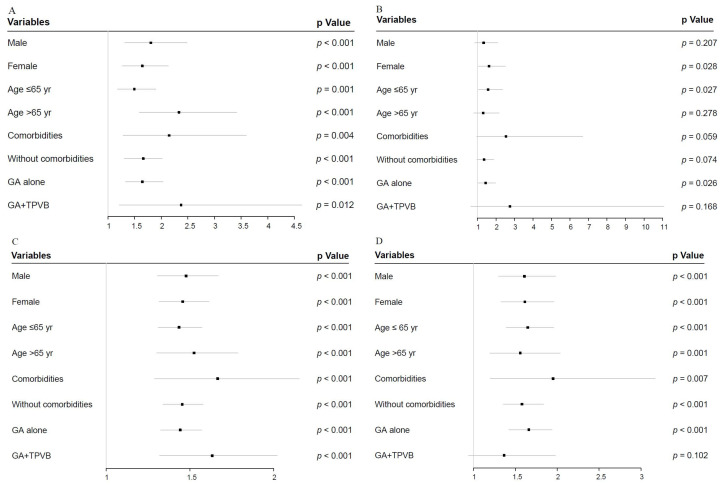

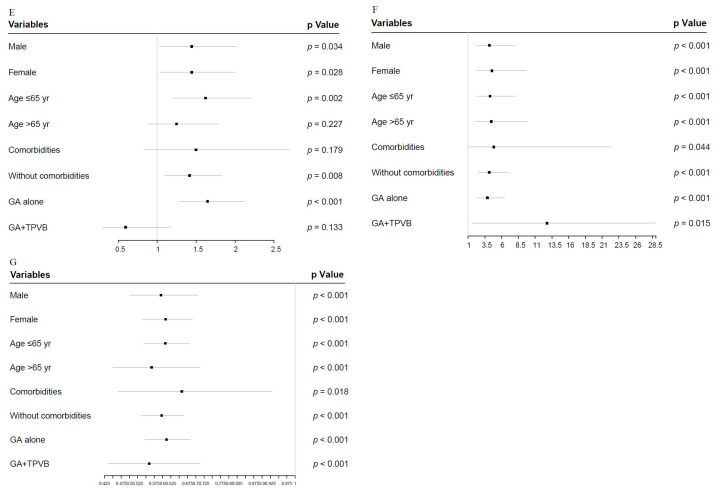
Forest plot of longer operative duration (>90 min) compared with shorter values (≤90 min), adjusted for covariates in sensitivity analysis. (**A**): Forest plot of hypoxemia. (**B**): Forest plot of delayed extubation. (**C**): Forest plot of PPCs. (**D**): Forest plot of PAL. (**E**): Forest plot of POAF. (**F**): Forest plot of transfusion. (**G**): Forest plot of early discharge. APOs: adverse perioperative complications; PPCs: postoperative pulmonary complications; PAL: prolonged air leakage; POAF: postoperative atrial fibrillation; TPVB: thoracic paravertebral blockade.

Given that 90 min may be the inflection point, we further evaluated the impact of longer operative duration (>90 min) on APOs and early discharge in four subgroup analyses, relative to shorter values (≤90 min) (Figure 3A–G). Compared with men, women with operative duration greater than 90 min had a higher rate of delayed extubation (OR = 1.335; 95% CI, 0.852–2.092; *p* = 0.207 vs OR = 1.625; 95% CI, 1.055–2.503; *p* = 0.028) (Figure 3B); however, the differences in longer operative duration by sex among other APOs and early discharge were not obvious.

Interestingly, compared with the reference category (>65 yr), individuals ≤65 yr with prolonged operative duration had a higher risk of delayed extubation (OR = 1.317; 95% CI, 0.801–2.166; *p* = 0.278 vs OR = 1.573; 95% CI, 1.054–2.350; *p* =0.027) and POAF (OR = 1.248; 95% CI, 0.871–1.789; *p* = 0.227 vs. OR = 1.620; 95% CI, 1.189–2.208; *p* = 0.002) (Figure 3B,E). More importantly, the longer operative duration in patients treated with general anesthesia (GA) plus TPVB did not affect the rates of delayed extubation (OR = 2.762; 95% CI, 0.652–11.703; *p* = 0.168 vs. OR = 1.436; 95% CI, 1.043–1.976; *p* = 0.026), PAL (OR = 1.366; 95% CI, 0.940–1.987; *p* = 0.102 vs. OR = 1.661; 95% CI, 1.422–1.939; *p* < 0.001), and POAF (OR = 0.594; 95% CI, 0.301–1.172; *p* = 0.133 vs. OR = 1.646; 95% CI, 1.278–2.121; *p* < 0.001) compared with patients treated with GA alone (Figure 3B–E).

## 4. Discussion

This study explored the continuous relationship of operative duration with a risk of the six most common APOs and the likelihood of early discharge among patients undergoing thoracoscopic lung cancer surgery. The risk of a “J-shaped” association with APOs was higher in patients with longer operative duration than those with shorter values, and conversely, an inverse “J-shaped” association was observed in early discharge. The 90 min procedure appears to be an inflection point for a sharp increase in APOs and a significant reduction in the likelihood of early discharge. Overall, these findings suggest that given the near exponential effect of operative duration on a risk of APOs, comprehensive preoperative evaluation and surgical planning should be performed to reduce operative duration.

Our study addressed an important knowledge gap in the medical literature regarding the ideal operative duration to limit the risk of APOs and promote early discharge during this surgical procedure. In previous studies, operative duration was not considered as the primary exposure and the interaction of multiple factors could not be completely excluded, leading to the lack of sufficient reliability. In addition, as a categorical variable, operative duration is beneficial to clinical application, but the data are underutilized, and continuous characteristics are lost [34]. Further, although it is widely recognized that operative duration is associated with increased morbidity and mortality in several surgical specialties [19,20,21,22,23,24], whether this relationship remains in this procedure, and the turning point leading to a significant increase in APOs and a lower rate of early discharge, is unknown [25,26]. In this cohort study, we used RCS functions to assess the continuous relationship of operative duration with a risk of APOs and early discharge, independent of potential covariates. Importantly, these findings showed that shortening the operative duration to less than 90 min may be an important adjustable factor to greatly reduce APOs and enhance early discharge.

The most common complication during OLV is hypoxemia, with a reported incidence of less than 4%, and possible mechanisms include the collapse of the nondependent lung and increased atelectatic areas in the dependent lung induced by forced an intrapulmonary shunt [5,13]. While the incidence of hypoxemia during OLV is low, the effects of progressive hypoxemia, especially in individuals with coexisting cardiovascular, cerebrovascular, or pulmonary disease, undoubtedly contribute to a greater risk of circulatory compromise [35]. Our study indicated that prolonged operative duration was significantly associated with an increased risk of hypoxemia, suggesting that shortening the operative duration may help prevent hypoxemia from occurring in patients with preoperatively existing cardiovascular complications.

The relationship between operative duration as a confounding factor and delayed extubation has been clearly articulated in the published literature [36,37]. This cohort study directly explored the continuous relationship between operative duration and the risk of delayed extubation and concluded that the risk increased in a time-dependent manner. Sensitivity analyses showed that women had a higher risk of delayed extubation than men did in the context of prolonged operative duration (>90 min), although sex was not traditionally recognized as an independent predictor [37]. Such results also suggest that we should pay more attention to the optimization of intraoperative management for female patients treated with longer operative duration. Interestingly, for delayed extubation, we found that the impact of longer operative duration was greater in patients aged ≤65 years than in older patients, potentially because younger patients were more sensitive to the effect of operative duration than their older counterparts.

Similar to what has been reported in published studies, our study demonstrated that PPCs and POAF were still the most common complications, along with arrhythmias, after thoracoscopic lung cancer surgery [8,9,31]. Thus, finding a protocol to reduce the incidence of PPCs and POAF remains a clinical concern for perioperative physicians. By using the Society of Thoracic Surgeons General Thoracic Surgery Database, Dexter and colleagues demonstrated that prolonged operative duration can increase the risk of PPCs, without a clear cutoff value that limits clinical utility [26]. The results of this study showed that limiting the operative duration to less than 90 min can greatly reduce the risk of PPCs and POAF, which has important clinical guiding significance given the exponential increase in the influence of longer operative duration on PPCs and POAF.

Additionally, according to our previous research and this cohort study, preoperative TPVB usage, as another modifiable measure, is associated with the reduced incidence of PPCs and POAF [38]. In the subgroup analysis, operative duration had no effect on the risk of delayed extubation and PAL in patients using TPVB, which means that TPVB usage may eliminate the effect of longer operative duration on the risk of delayed extubation and PAL. TPVB’s lowering delayed extubation may be due to reduced intraoperative opioid doses, improved postoperative pain control, and improved patient tolerance to tracheal and thoracic tubes [39,40,41]. The underlying biological and mechanistic mechanisms by which TPVB reduces the incidence of PAL remain unclear.

PAL occurs frequently after thoracic surgery because of a lung parenchymal injury, and it was associated with prolonged chest tube drainage and LOS, a poor quality of life, and a high financial expense [10,42]. The relationship between longer operative duration and the risk of PAL has not been generally recognized by other investigators [10,11,26], but this study is the first to report a significant increase in the risk of PAL with increasing operative duration. In contrast to other studies, the results found in this study could be explained by the different types of surgical inclusion and the large sample size analyzed. Therefore, for patients at a high risk of PAL, reducing the operative duration may be a feasible and adjustable method, especially if the operative duration is controlled within 90 min. As we expected, operative duration was significantly positively correlated with transfusion, which is reasonably explained by the fact that a longer operative duration means that it may be a more complex or difficult anatomical marker, leading to an increased likelihood of transfusion. Similarly, prolonged operative duration indirectly affects LOS or early discharge by increasing the rates of perioperative complications, which is consistent with other reports [26,38]. This study also found that within 90 min, the likelihood of early discharge precipitously decreased as the duration of surgery increased.

## 5. Strengths and Limitations

Our study has several important strengths. This was a study based on a large sample database collected prospectively, and the statistical methods and main outcomes were developed and completed before the start of this trial. To the best of our knowledge, this study is the first to use the RCS functions to investigate the continuous relationship of operative duration with a risk of the six most common APOs and early discharge in thoracoscopic lung cancer surgery. Likewise, several limitations are among our research. First, as a monocentric retrospective study, it has inherent design biases. Second, because of the limited granularity of postoperative care data, other surgical complications that may be affected by operative duration, such as the timing of chest tube removal and intensive care unit (ICU) admission [26], could not be included in this study. Third, the continuous relationship between operative duration and patients’ long-term outcomes needs further investigation.

## 6. Conclusions

This retrospective cohort study included 12,392 patients who underwent this surgical treatment, and the study explored the continuous relationship of operative duration with a risk of the six most common APOs and early discharge. The results of this study showed that the risk of APOs exponentially increases as the operative duration exceeds 90 min, accompanied by a significant decrease in the likelihood of early discharge, indicating that shortening the operative duration to less than 90 min may be an important adjustable factor to greatly reduce APOs and accelerate early discharge. Our findings have important and meaningful implications for risk predictions and clinical interventions, and early rehabilitation, for APOs.

## Figures and Tables

**Table 1:** Perioperative characteristics stratified by operative duration.

	<60 min	60–120 min	120–180 min	180–240 min	≥240 min
Variables ^a^	(*n* = 1336)	(*n* = 8365)	(*n* = 2322)	(*n* = 307)	(*n* = 62)
Age, years	56.4 ± 11.2	57.5 ± 10.6	59.6 ± 10.2	61.6 ± 9.6	62.6 ± 10.1
Sex					
Male sex	404 (30.2)	3133 (37.5)	1145 (49.3)	199 (64.8)	36 (58.1)
Female sex	932 (69.8)	5232 (62.5)	1177 (50.7)	108 (35.2)	26 (41.9)
BMI, kg/m^2^	22.7 ± 2.8	23.2 ± 3.0	23.7 ± 3.1	23.6 ± 2.9	23.5 ± 3.8
ASA grade					
I	151 (11.3)	777 (9.3)	161 (6.9)	17 (5.5)	3 (4.8)
II	1058 (79.2)	6767 (80.9)	1933 (83.2)	254 (82.7)	49 (79.0)
III/IV	127 (9.5)	821 (9.8)	228 (9.9)	36 (11.7)	10 (16.1)
Comorbidity					
Hypertension	76 (5.7)	609 (7.3)	154 (6.6)	30 (9.8)	3 (4.8)
Diabetes mellitus	26 (1.9)	275 (3.3)	96 (4.1)	17 (5.5)	2 (3.2)
Coronary artery disease Stroke/TIA	6 (0.4) 2 (0.1)	46 (0.5) 27 (0.3)	11 (0.5) 4 (0.2)	3 (1.0) 1 (0.3)	0 (0) 0 (0)
FEV_1_/FVC, %	101.6 ± 7.1	101.3 ± 7.7	100.8 ± 8.5	100.2 ± 9.2	97.3 ± 11.5
DLCO%	94.8 ± 14.4	94.9 ± 15.4	93.3 ± 15.6	90.7 ± 18.3	87.0 ± 19.7
Chemoradiotherapy	2 (0.1)	22 (0.3)	7 (0.3)	5 (1.6)	1 (1.6)
Tumor size, cm	1.4 ± 0.8	1.7 ± 1.0	2.0 ± 1.2	2.2 ± 1.6	2.6 ± 1.7
Advanced clinical T stage (T ≥ 2)	63 (4.7)	772 (9.2)	344 (14.8)	58 (18.9)	17 (27.4)
Pleural adhesions	6 (0.4)	120 (1.4)	104 (4.5)	32 (10.4)	9 (14.5)
Type of anesthesia GA alone	1105 (82.7)	7163 (85.6)	1989 (85.7)	268 (87.3)	54 (87.1)
GA plus TPVB	231 (17.3)	1202 (14.4)	333 (14.3)	39 (12.7)	8 (12.9)
Type of resection					
Segmentectomy resection	482 (36.1)	1752 (20.9)	360 (15.5)	44 (14.3)	7 (11.3)
Lobectomy resection	854 (63.9)	6613 (79.1)	1962 (84.5)	263 (85.7)	55 (88.7)
Surgical procedure					
VATS	1296 (97.0)	7939 (94.9)	2271 (97.8)	304 (99.0)	62 (100.0)
RATS	40 (3.0)	426 (5.1)	51 (2.2)	3 (1.0)	0 (0)
Location of resection					
Left resection	546 (40.9)	3191 (38.1)	873 (37.6)	125 (40.7)	28 (45.2)
Right resection	790 (59.1)	5174 (61.9)	1449 (62.4)	182 (59.3)	34 (54.8)
Clinical nodal involvement	29 (2.2)	334 (4.0)	201 (8.7)	40 (13.0)	8 (12.9)
Lymph nodes calcification	31 (2.3)	319 (3.8)	175 (7.5)	54 (17.6)	11 (17.7)
Hypoxemia	26 (1.9)	284 (3.4)	134 (5.8)	22 (7.2)	4 (6.5)
Delayed extubation	12 (0.9)	101 (1.2)	57 (2.5)	12 (3.9)	7 (11.3)
PPCs	269 (20.1)	2727 (32.6)	948 (40.8)	138 (45.0)	33 (53.2)
PAL	60 (4.5)	578 (6.9)	269 (11.6)	60 (19.5)	17 (27.4)
POAF	12 (0.9)	207 (2.5)	87 (3.7)	24 (7.8)	10 (16.1)
Transfusion	5 (0.4)	43 (0.5)	47 (2.0)	20 (6.5)	11 (17.7)
Early discharge	435 (32.6)	1346 (16.1)	266 (11.5)	21 (6.8)	4 (6.5)

^a^ Continuous data are shown as mean ± standard deviation (SD) and categoric data as a number (%). BMI: body mass index; ASA: American Society of Anesthesiology; TIA: transient cerebral ischemic attack; FEV_1_: forced expiratory volume in 1 s; FVC: forced vital capacity; DLCO: diffusion capacity for carbon monoxide. VA/RATS: video-assisted/robotic-assisted thoracoscopic surgery; TPVB: thoracic paravertebral blockade; PPCs: postoperative pulmonary complications; PAL: prolonged air leakage; POAF: postoperative atrial fibrillation.

**Table 2:** Adjusted odds ratio (OR) for perioperative outcomes stratified by operative duration (vs. <60 min).

	60–120 min		120–180 min		180–240 min		≥240 min	
Outcomes ^a^	OR (95% CI)	*p*-Value	OR (95% CI)	*p*-Value	OR (95% CI)	*p*-Value	OR (95% CI)	*p*-Value
Hypoxemia	1.628 (1.080–2.454)	0.020 ^b^	2.574 (1.670–3.968)	<0.001 ^b^	3.111 (1.723–5.620)	<0.001 ^b^	2.618 (0.856–8.006)	0.091
Delayed extubation	1.209 (0.661–2.210)	0.538	2.182 (1.160–4.103)	0.016 ^b^	2.932 (1.282–6.704)	0.011 ^b^	8.727 (3.225–23.616)	<0.001 ^b^
PPCs	1.822 (1.579–2.102)	<0.001 ^b^	2.391 (2.037–2.806)	<0.001 ^b^	2.666 (2.045–3.475)	<0.001 ^b^	3.631 (2.157–6.113)	<0.001 ^b^
PAL	1.366 (1.038–1.797)	0.026 ^b^	1.987 (1.480–2.669)	<0.001 ^b^	2.925 (1.953–4.380)	<0.001 ^b^	4.082 (2.137–7.801)	<0.001 ^b^
POAF	2.566 (1.426–4.616)	0.002 ^b^	3.272 (1.773–6.036)	<0.001 ^b^	5.647 (2.751–11.590)	<0.001 ^b^	12.839 (5.195–31.730)	<0.001 ^b^
Transfusion	1.350 (0.533–3.420)	0.527	4.838 (1.909–12.265)	0.001 ^b^	13.844 (5.092–37.637)	<0.001 ^b^	41.063 (13.459–125.285)	<0.001 ^b^
Early discharge	0.465 (0.407–0.531)	<0.001 ^b^	0.350 (0.293–0.418)	<0.001 ^b^	0.217 (0.137–0.346)	<0.001 ^b^	0.227 (0.081–0.638)	0.005 ^b^

^a^ Multivariate logistic regression model using the forward selection strategy. ^b^ Statistically significant (*p* < 0.05). OR: odds radio; CI: confidence interval; PPCs: postoperative pulmonary complications; PAL: prolonged air leakage; POAF: postoperative atrial fibrillation.

## Data Availability

Our research team could provide original data under reasonable request and with permission from Shanghai Chest Hospital.

## Supplementary Materials

The following supporting information can be downloaded at https://www.mdpi.com/article/10.3390/cancers15020371/s1: Figure S1: Patient flowchart.
