# Supplementary figures and images for: Continuous Relationship of Operative Duration with Risk of Adverse Perioperative Outcomes and Early Discharge Undergoing Thoracoscopic Lung Cancer Surgery
Source: Cancers (Basel). 2023 Jan 6;15(2):371. doi: 10.3390/cancers15020371 (PMC9856387; doi:10.3390/cancers15020371)

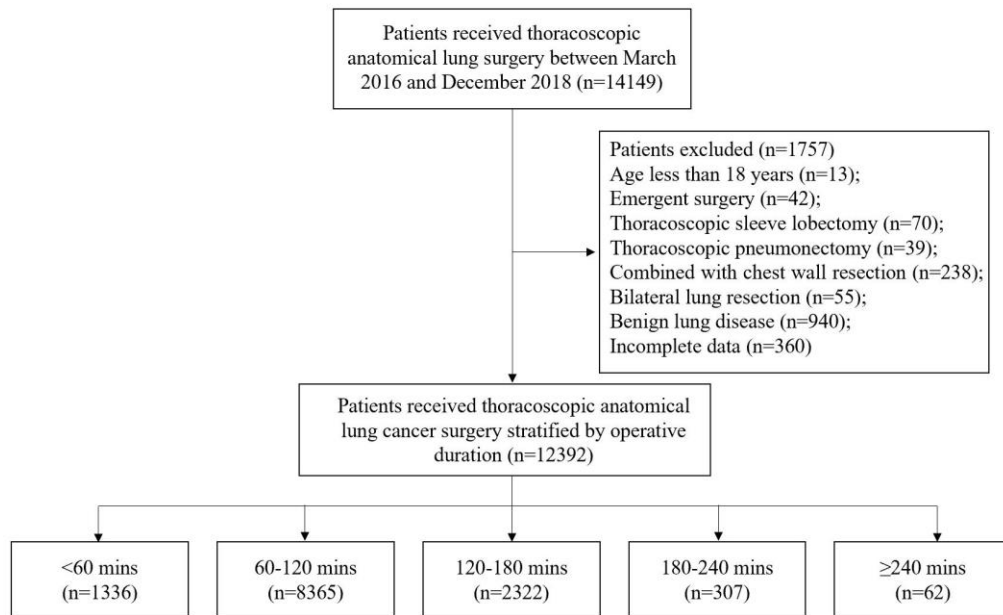

Supplementary Figure S1 Patient flowchart.

Supplement: Supplementary file 1 [file cancers-15-00371-s001.zip › cancers-2035189-supplementary.pdf]
